# The Gulf Stream

**Published:** 1892-09

**Authors:** 


### THE GULF STREAM.

It has been truly said that were it not for the movement of the tides, which constantly agitate the vast waters which encircle the globe, life upon our planet would be impossible. The Gulf Stream in its everlasting sweep, is one of the great factors in producing climatic conditions suited to the higher forms of animal existence.

The currents of the ocean are the great transporters of the sun's heat from the torrid zone to temper the climate of the polar regions, says John E. Pillsbury in the *Century.* It is argued by some that such a stupendous change as that which occurred in Europe and America at the time of the glacial period was caused simply by a deflection in the currents in the northern hemisphere, whereby its share of tropical heat was partly diverted toward the south. In the three great oceans, the Atlantic, the Pacific, and the Indian, there is to be found a similar circulation—a general westerly movement in the tropics, a flow toward the poles along the eastern shores of the continents, an easterly set in the temperate zones, and a current toward the equator along the western shores. This system thus becomes a grand circular movement, some parts being very slow, but still quite constant, and other parts very swift. There are offshoots here and there, due to local causes, and perhaps in the slowly moving current there may be a temporary interruption, but, taken as a whole, the movement is continuous.

The part of this circulation flowing along the eastern coast of the United States is the greatest of all these currents, and, in fact, is the most magnificent of all nature's wonders. This is the Gulf Stream. The name Gulf Stream was first suggested by Benjamin Franklin because it comes from the Gulf of Mexico. While it is a portion of the grand scheme of ocean circulation, and the Gulf of Mexico is in reality only a stopping place, as it were, for its waters, the name is generally applied to the current when it reaches the straits of Florida, north of Cuba. In the large funnel shaped opening towards the Gulf of Mexico the current at first is variable in direction and velocity, but by the time Havana is reached it has become a regular and steady flow. As it rounds the curve of the Florida shore the straits contract, and the water then practically fills the banks from shore to shore and reaches almost to the bottom, which is at this point about 3,000 feet deep. As it leaves the straits of Florida its course is about north, but it gradually changes in direction, following approximately the curve of 100 fathoms deep until it reaches Cape Hatteras. From this point it starts on its course to Europe. It has lost something in velocity as well as in temperature, and as it journeys to the eastward it gradually diminishes in both, until it becomes a gentle flow as it approaches Europe.

People think the Mississippi river a grand river, and it is so in truth, as far as land rivers go; but great as it is it would require 2,000 such rivers to make one gulf stream. The great ocean river is an irresistible flood of water, running all the time, winter and summer, and year after year. It is as difficult for the mind to grasp its immensity as it is to realize the distance of the nearest stars. At its narrowest part in the straits of Florida it is thirty-nine miles wide, has an average depth of 2,000 feet, and a velocity at the axis—the point of fastest flow—of from three to more than five miles per hour. To say that the volume in one hour's flow past Cape Florida is 90,000,000,000 tons in weight does not convey much to the mind. If we could evaporate this one hour's flow of water and distribute the remaining salt to the inhabitants of the United States, every man, woman, and child would receive nearly sixty pounds.

It is curious to note in the history of the Gulf Stream how great its influence has been on the fortunes of the new world. Before the discovery of America strange woods and fruits were frequently found on the shores of Europe and off-lying islands. Some of these were seen and examined by Columbus, and to his thoughtful mind they were confirming evidence of the fact that strange lands were not far to the westward. These woods were carried by the gulf stream and by the prevailing winds from the American continent, so that in part the gulf stream is responsible for the discovery of the new world. Ponce de Leon, while on his famous search for the fountain of youth, made the discovery of this more practically beneficial phenomenon. The whalers of New England were the first to gain a fairly accurate knowledge of the limits of the current between America and Europe by following the haunts of the whales, which were found north of one line and south of another, but never between the two. This, they reasoned, was the gulf stream current. Benjamin Franklin received this information from the whalers, and published it on a chart for the benefit of the mail packets plying between England and the colonies. The chart was first issued about 1770, but was not accepted by the English captains. Before it came to be generally known and used the trouble between England and the colonies had begun, and Franklin, knowing the advantage the knowledge would be to the British officers, suppressed it all he could until hostilities ceased.

